# Pressure-Driven Helium Insertion for Structural Stability of CH_3_NH_3_PbBr_3_ Hybrid Perovskites

**DOI:** 10.1021/acs.chemmater.5c03238

**Published:** 2026-03-31

**Authors:** Nicholas J. Weadock, Willis Holle, Kiley Mayford, Stefano Racioppi, Anukriti Ghimire, Dylan M. Ladd, Changyong Park, Eva Zurek, Michael F. Toney, Frank Bridges, Shanti Deemyad

**Affiliations:** † Materials Science and Engineering, University of Colorado, Boulder, Colorado 80309, United States; ‡ Department of Physics and Astronomy, 7060 University of Utah, Salt Lake City, Utah 84112, United States; § Physics Department, 8787 University of California, Santa Cruz, California 95064, United States; ∥ Department of Chemistry, State University of New York at Buffalo, Buffalo, New York 14260, United States; ⊥ Department of Materials Science and Metallurgy, University of Cambridge, Cambridge CB30FS, U.K.; # High Pressure Collaborative Access Team (HPCAT), X-ray Science Division, 1291 Argonne National Laboratory, Lemont, Illinois 60439, United States; ∇ Department of Chemical and Biological Engineering, University of Colorado, Boulder, Colorado 80309, United States; ° Renewable and Sustainable Energy Institute (RASEI), University of Colorado, Boulder, Colorado 80309, United States

## Abstract

Organic–inorganic metal halide perovskites (MHPs) have garnered significant attention due to their outstanding performance in optoelectronic devices, but they are prone to degradation through a variety of pathways. High-pressure studies are being used to understand the mechanical and structural stability of MHPs and investigate new methods for improving these. In this study, we map the high-pressure, low-temperature structural phase diagram of CH_3_NH_3_PbBr_3_ (MAPbBr_3_) between 15–300 K and up to 1.5 GPa. We first compare the temperature and pressure effects on the global and local structures of MAPbBr_3_ using synchrotron X-ray diffraction (XRD) and extended X-ray absorption fine structure (EXAFS), respectively, and find evidence of PbBr_6_ octahedral distortion. Our results also suggest that He inserts into the MAPbBr_3_ structure. The thermodynamics of He insertion into MAPBHe_*x*_ are calculated to be plausible at room temperature for *x* ≤ 1 and *P* = 1–3 GPa and shown to stabilize higher-symmetry phases. This phenomenon increases the fitted bulk modulus of MAPbBr_3_. Structural stabilization by inert atom insertion is proposed as a method to improve the mechanical robustness of MHPs and other soft hybrid materials.

## Graphical abstract

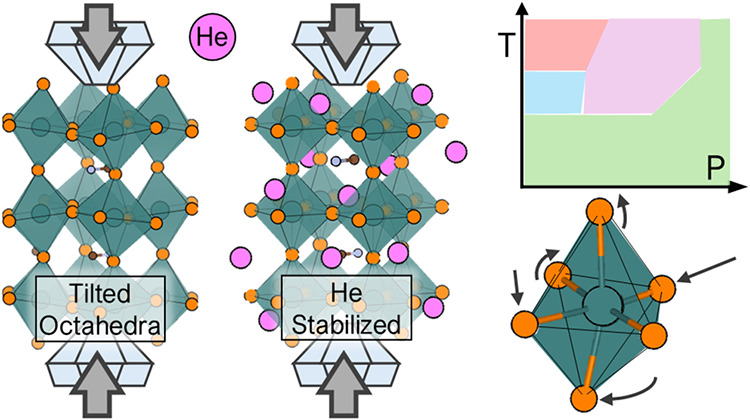

## Introduction

Solar energy is the most abundant and accessible renewable resource on Earth, offering a clean and sustainable solution to meet growing global energy demands. Harnessing this potential requires materials that can efficiently capture and convert sunlight into electricity. Among the most promising candidates are hybrid organic–inorganic lead halide perovskite semiconductors (LHPs) with the chemical formula ABX_3_, where A = Cs^+^, methylammonium (CH_3_NH_3_^+^ = MA^+^), or formamidinium (CH­(NH_2_)_2_^+^ = FA^+^), B = Pb^2+^ or Sn^2+^, and X = Cl^–^, Br^–^, or I^–^. LHPs are structurally analogous to classic oxide perovskites such as CaTiO_3_ and MgSiO_3_ and benefit from significant structural flexibility that allows for diverse ionic substitutions and alloying. LHP-based solar cells have rapidly achieved power conversion efficiencies exceeding 26%.^1^ This high efficiency is due to the excellent optoelectronic properties of LHPs including strong optical absorption, long carrier lifetimes, and tunable band gaps.^2^ These properties are also favorable for several other applications including high-energy X-ray and γ-ray detectors, light-emitting diodes, and optospintronic devices.^3–5^

However, despite their rapid progress, real-world deployment remains limited by a few critical challenges, one of which is the loss of mechanical stability between the LHP active layer and charge transport layers in LHP-based devices due to interfacial strains generated by differences in the coefficient of thermal expansion (CTE) between layers, residual stresses, and structural phase transitions of the LHP.^6,7^ Depending on the LHP stoichiometry, these structural phase transitions occur at temperatures within the temperature range for device operation, compounding the CTE mismatch effects.^8–10^ One of the prototypical LHPs, MAPbBr_3_, undergoes temperature-driven symmetry-lowering transitions from cubic to tetragonal (236 K) to orthorhombic (147 K) primarily governed by tilting of the PbX_6_ octahedra.^11^

There has been a significant research effort to stabilize the preferred high-symmetry cubic phase for LHP devices. One initial approach uses alloying to manipulate the Goldschmidt Tolerance Factor (GTF), an empirical rule that predicts cubic-phase stability by the ratio of the A,B and X-site ionic radii.^12,13^ For this alloying approach, care must be taken to ensure compositional homogeneity and maintain the desired optical bandgap.^9,14^ Alloying also provides the benefit of shifting the structural phase transition temperatures, as expected when those of the alloy end members differ.^9,14–16^

A second approach, at first glance less practical than alloying, is to apply external pressure to the LHP as a way to extend the stability of the cubic phase. High-pressure X-ray diffraction (XRD) studies have revealed pressure-induced octahedral tilting patterns that differ from those observed at low temperatures.^17–24^ Weak interoctahedral interactions, combined with interatomic potentials that have large anharmonic coefficients, drive the thermally induced structural phase transitions, as octahedral tilts are dynamically unlocked. Applying large external pressures can “lock in” the tilts, accessing the low-temperature, ambient-pressure crystal structures at higher temperatures.^25–27^

A third, more recent approach involves the replacement of both an A^+^ and X^–^ site with organochalcogenide zwitterionic RCh ligands, where RCh = CYS or SeCYS (csyteamine; ^+^NH_3_(CH_2_)_2_S^–^ or selenocysteamine; ^+^NH_3_(CH_2_)_2_SE^–^).^28,29^ The RCh ligand covalently bonds the organic A-site cation to the chalcogenide X-site anion, introducing structural rigidity against octahedral tilting and pressure-induced phase transitions. The *R*3̅*c* structure is preserved up to 40 GPa (with Ne pressure medium) with no signs of any amorphization that occurs for the prototypical MAPbX_3_ LHPs.^30^

In all approaches, however, changes in the local structure have not been thoroughly characterized. It is well established that, despite XRD results showing the presence of highly crystalline “global” structures, the presence of dynamic and static domains with lower symmetry and lower dimensionality determines many of the relevant optoelectronic properties of single-phase, unalloyed 3D LHPs.^31–35^ The local structure manifests in the diffuse background of X-ray and neutron total scattering measurements and is interpreted with pair-distribution function (PDF) analysis, structural modeling, or by direct comparison to molecular dynamics simulations. Additional dynamic correlations are probed with inelastic scattering and spectroscopy techniques.^11,36^

First, we report a comprehensive study of the pressure and temperature phase behavior of the MAPbBr_3_. The crystal structures of MAPbBr_3_ at high pressures and ambient temperature have been explored by several groups; most pressure–temperature (*P–T*) phase diagrams have been derived from dielectric or spectroscopic measurements.^37,38^ Detailed crystallographic studies, particularly those that resolve local structural changes across phase boundaries, are still lacking. For a recent study using neutron and X-ray diffraction to map the (*P–T*) phase diagram for MAPbI_3_, see Marin-Villa et al.^39^

The high-pressure, low-temperature *P–T* phase diagram (0–1.5 GPa, 15–300 K) we report is complemented by a study of the high-pressure local structure as determined through extended X-ray absorption fine structure (EXAFS) measurements. This builds on work previously reported by some of the authors that combined EXAFS, single-crystal X-ray diffraction (SCXRD), and *ab initio* molecular dynamics (AIMD) to investigate the nature of disorder in APbBr_3_ (A = MA^+^, Cs^+^, and FA^+^).^11,36^ These previous results revealed that thermal fluctuations significantly influence the local, asymmetric Pb–Br bonding environment. They proposed that a single-site Pb–Br anharmonic potential is the origin of this thermal disorder. Furthermore, changes in the transience and strength of the H···Br bond, respectively, increase and decrease the Pb–Br interatomic potential in MAPbBr_3_ and FAPbBr_3_.

In this work, we show that while pressure reduces the lattice parameter, the average Pb–Br bond length is largely unaffected. Instead, the width of the Pb–Br PDF increases, suggesting that the PbBr_6_ octahedra themselves become distorted with pressure, yielding a larger range of individual Pb–Br bond lengths. PDF in this work refers to the pair-distribution function obtained through EXAFS analysis and not the more common X-ray total scattering approach. This result is consistent with recent modeling of high-pressure X-ray total scattering experiments on the analogous all-inorganic LHP, CsPbBr_3_.^40^ Large changes in the Br–Br total scattering PDF are observed with increasing pressure; however, the behavior of the Pb–Br and Pb–Pb PDFs remains comparatively unchanged. The authors conclude that this behavior indicates distortion of individual PbBr_6_ octahedra without disruption of the lead substructure. Our results expand on this study by investigating the high-pressure local structure in hybrid organic LHPs in the high-temperature cubic phases, where dynamic local order is known to exist.

Finally, we propose an alternative method to improve cubic-phase stability. The variation in reported bulk modulus for MAPbBr_3_ suggests that He, when used as a pressure-transmitting medium, may insert into the MAPbBr_3_ lattice and influence its structural response. Previous theoretical work by some of these authors identified that He can be inserted into several ReO_3_-type perovskites at He pressures on the order of 10 GPa.^41^ He insertion has been experimentally demonstrated in perovskites,^42–44^ glasses,^45^ and clathrates.^46–48^ A recent study demonstrated that He incorporation in the A-site vacant perovskite ScF_3_ maintains the cubic structure under compression up to 5 GPa; without He, there is a structural phase transition to a rhombohedral phase at 0.7 GPa.^63^ We explore the possibility that He insertion can occur at high pressures through density functional theory (DFT) and AIMD calculations. Our results suggest that up to one formula unit of He is thermodynamically metastable and dynamically stable in the MAPbBr_3_ structure at 1–3 GPa and ambient temperature. Furthermore, at 300 K and 0.3 GPa, the MAPbBr_3_He_*x*_ structure calculated from AIMD shows a higher symmetry than that of the pure MAPbBr_3_ structure. These results point to a new method of inert atom or molecule insertion as an alternative mechanism for stabilizing the cubic phase and improving mechanical stability, offering a novel path for designing more robust hybrid materials at ambient conditions.

## Methods

### Sample Preparation

#### MAPbBr_3_ Sample Preparation

MAPbBr_3_ single crystals were grown using the established inverse temperature crystallization method.^49^ *N*,*N*-dimethylformamide (≥99.8% anhydrous), MABr (≥99% anhydrous), and PbBr_2_ (≥98%) were purchased from Sigma-Aldrich. In a nitrogen glovebox, a stoichiometric ratio of 1:1 MABr/PbBr_2_ precursor salts were dissolved in DMF and stirred to obtain a clear 1 M MAPbBr_3_ stock solution. The solution was filtered through a 0.2 μm PTFE filter and sealed in a clean vial for growth. The growth vial was removed from the glovebox and partially submerged in an oil bath on a hot plate. The temperature was gradually increased from ambient to 90 °C at a rate of 5 °C/h. MAPbBr_3_ single crystals 0.5–2 mm in size were harvested from the hot solution, gently patted dry, and stored in a nitrogen drybox until use.

High-quality MAPbBr_3_ single crystals were visually identified and then gently crushed and ground to a fine powder with an agate mortar and pestle. The powders were then gently annealed at 90 °C for 15 min in flowing nitrogen to reduce defects introduced by grinding. The annealed powders were passed through a 400 mesh sieve to isolate grains of 32 μm and smaller. The quality of the initial powder was inspected with X-ray diffraction using a Rigaku SmartLab 9 kW diffractometer using Cu Kα radiation (λ = 1.54 Å). The powders were measured in Bragg–Brentano geometry from 10 to 80° 2θ at 5°/min. The Kα splitting is visibly distinct at 30° 2θ; these narrow peaks suggest large crystallites (>200 nm) and minimal defects. See Figure S1 in the SI for the XRD results.

### High-Pressure Synchrotron X-ray Characterization

#### Diamond Anvil Cell Preparation and X-ray Diffraction Measurements

Symmetric diamond anvil cells (DACs) with 350 μm culet sizes were used to generate high pressures. Beryllium gaskets were employed and loaded with lithium fluoride (LiF) as the pressure-transmitting medium (PTM). Both beryllium and LiF were selected for their low atomic numbers to minimize X-ray absorption, enabling effective extended X-ray absorption fine structure (EXAFS) measurements through the gasket, while X-ray diffraction (XRD) measurements were performed through the diamonds. The cryostat featured four opposing windows and could rotate by 90°, allowing for both XRD and EXAFS measurements at each pressure and temperature condition. After initial compression, the LiF in the gasket was drilled with a laser to create a slit (≈30 × 110 μm^2^), which was then filled with powdered MAPbBr_3_. Pressure was determined using ruby fluorescence with additional calibration provided by the known equation of state for LiF. A double-membrane assembly was utilized for *in situ* pressure adjustments during the experiments. Diffraction data were collected at the 16-BM-D beamline at the Advanced Photon Source (APS) using an X-ray wavelength of 0.4133 Å. After initial pressurization, the DACs were cooled to low temperatures. Diffraction measurements were carried out between 15 and 300 K with small pressure intervals. Three spots within the pressure chamber were used for data collection, and the background was measured from LiF regions devoid of the sample to exclude peaks unrelated to the sample. See Figure S2 for a schematic of the experimental setup. Sequential EXAFS and XRD were performed only for a selected number of pressure and temperature points.

#### High-Pressure X-ray Absorption Spectroscopy Analysis

The Pb L_III_ data were reduced using the Real Space X-ray Absorption Package (RSXAP).^50^ The pre-edge backgrounds were removed via fitting to Chebyshev polynomials using least-squares. The EXAFS function χ­(*E*), defined as a modulation of the absorption coefficient as a function of energy, is given by μ­(E) = μ_0_(*E*) (1 + χ­(*E*)), where μ­(*E*) is the measured absorption coefficient and μ_0_(*E*) is a smooth background function. A spline function is used to model μ_0_ in order to extract the EXAFS quantity χ­(*E*), which is then converted to χ­(*k*). Finally, χ­(*k*) is fast Fourier transformed into real space using a Fourier Transform (FT) range from 4 to 9.7 Å^–1^. The upper end of the FT range is limited by the presence of the Br K edge.

The Pb–Br peak was fit to a Pb–Br PDF calculated using FEFF7;^51^ from our earlier work^11,36^ it was clear that the asymmetry parameter *C*_3_ had to be included at room temperature. The EXAFS equation used here is the same as our previous work with the kurtosis parameter *C*_4_ set to 0

1 $$\begin{array}{l} k\chi_{s}\left(k,r\right)=A_{1}\sin\left(2\mathrm{kr}-\frac{4}{3}C_{3}k^{3}+\phi \left(k\right)\right)\\ A_{1}=\frac{NS_{0}^{2}}{r^{2}}F\left(\pi ,k\right)e^{-2r/\lambda \left(k\right)}\\ k\chi_{\mathrm{Pb}\text{−}\mathrm{Br}}\left(k\right)=\int_{0}^{\infty }g_{\mathrm{Pb}\text{−}\mathrm{Br}}\left(r,R\right)k\chi_{s}\left(k,r\right)dr \end{array}$$

with *N* the number of Br neighbors, S_0_^2^ the amplitude reduction factor, *F* the backscattering amplitude of the photoelectron wave, λ the mean free path, ϕ­(*k*) is a known phase factor, calculated using FEFF7,^51^ arising from the excited and backscattering atoms, and *C*_3_ is the skewness of the PDF, a measure of the asymmetry. The term e^–2*r*/λ(*k*)^ is often combined with *F*. Finally, *g*_Pb–Br_(*r*, *R*) is the PDF centered at *R*; the variance of this function, σ^2^, is usually called the Debye–Waller factor.

However, because of the relatively low signal-to-noise quality of the data, we could not get a robust fit, allowing both *C*_3_ and *R* to vary freely. Instead, we fixed *C*_3_ to the values obtained earlier^11^ and only allowed σ^2^ and *R* to vary for the pressure measurements in order to obtain a plausible fit.

### Computational Details

We performed periodic Density Functional Theory (DFT) calculations using VASP, version 6.4.2. The opt-B88-vdw exchange-correlation functional, which accounts for dispersion interactions, was employed in combination with the projected augmented-wave (PAW) method, with a cutoff of 1100 eV. The *k*-point meshes were generated using the Γ-centered Monkhorst–Pack scheme, and the number of divisions along each reciprocal lattice vector was selected so that the product of this number with the real lattice constant was greater than or equal to 50 Å. For the geometry optimizations, the accuracy of the energy convergence was set to 10^–6^ eV and the norms of all the forces calculated during the relaxations were smaller than 10^–3^(eV/Å). The Gaussian smearing method was adopted in the geometry optimizations with a σ value of 0.02. Phonons in the harmonic approximation were determined with the Phonopy package using supercells equal to 5 × 5 × 3 for He and to 2 × 1 × 2 for MAPbBr_3_He_*x*_ (*x* = 0, 0.5, and 1), based on the conventional unit cells so that all cell parameters *a*,*b*, and *c* were greater than 10 Å. A finite displacement of 0.003 Å was used, and the accuracy of the energy convergence was set to 10^–8^eV. The enthalpies and Gibbs free energies for the reaction MAPbBr_3_ + *x*He → MAPbBr_3_He_*x*_ were calculated for the pressure range from 1 to 3 GPa and for the temperature range from 0 to 300 K. In the helium uptake reaction, *x* represents the helium molar fraction inserted per MAPbBr_3_ unit and takes the values of 0.5 and 1. MAPbBr_3_ and MAPbBr_3_He were found to be dynamically stable with the space group *Pnma*, while MAPbBr_3_He_0.5_ lowers the symmetry to *P2*_1_/*c* (structures reported in the Supporting Information). To calculate the enthalpy and Gibbs free energy of helium, we considered the most stable crystalline form between 0 and 1 GPa, the hexagonal close-packed *P6*_3_ */mmc*.

Molecular dynamics using the isothermal–isobaric (NPT) ensemble and the Langevin thermostat at *T* = 300 K (a Fermi smearing was employed) and at *P* = 0.3 GPa were performed for MAPbBr_3_ and MAPbBr_3_He with VASP. In this case, the PBE functional was used in combination with the D3-BJ Grimme dispersion correction on 2 × 2 × 2 supercells, equal to 400 atoms for MAPbBr_3_He. The duration of the simulation was set to 5 ps with 1 fs time steps. The projected augmented-wave (PAW) method with a cutoff of 900 eV was used, and the accuracy of the energy convergence was set to 10^–5^ eV.^52^

## Results

### Long-Range Structure from X-ray Diffraction

Figure 1a,b presents the XRD data collected across a range of pressures and temperatures. Figure 2 shows the pressure–temperature (*P–T*) phase diagram for MAPbBr_3_ constructed in this study, alongside previously reported data from Jaffe et al., and Wang et al.^24,53^ Four distinct structural phases: cubic I (*Pm*3̅*m*), cubic II (*Im*3̅), tetragonal *I*4/*mcm*, and orthorhombic *Pnma*, are identified, in agreement with the earlier literature. At ambient pressure, we observe the cubic-to-tetragonal transition around 236 K and the tetragonal-to-orthorhombic transition near 147 K, consistent with prior reports. Figure 1b also shows the evolution of the Bragg peak breadths during the measurement; before compression at ambient temperature, the Bragg peaks are very sharp and broaden with compression. We expect that this broadening comes from the introduction of crystalline defects.

**Figure 1:**
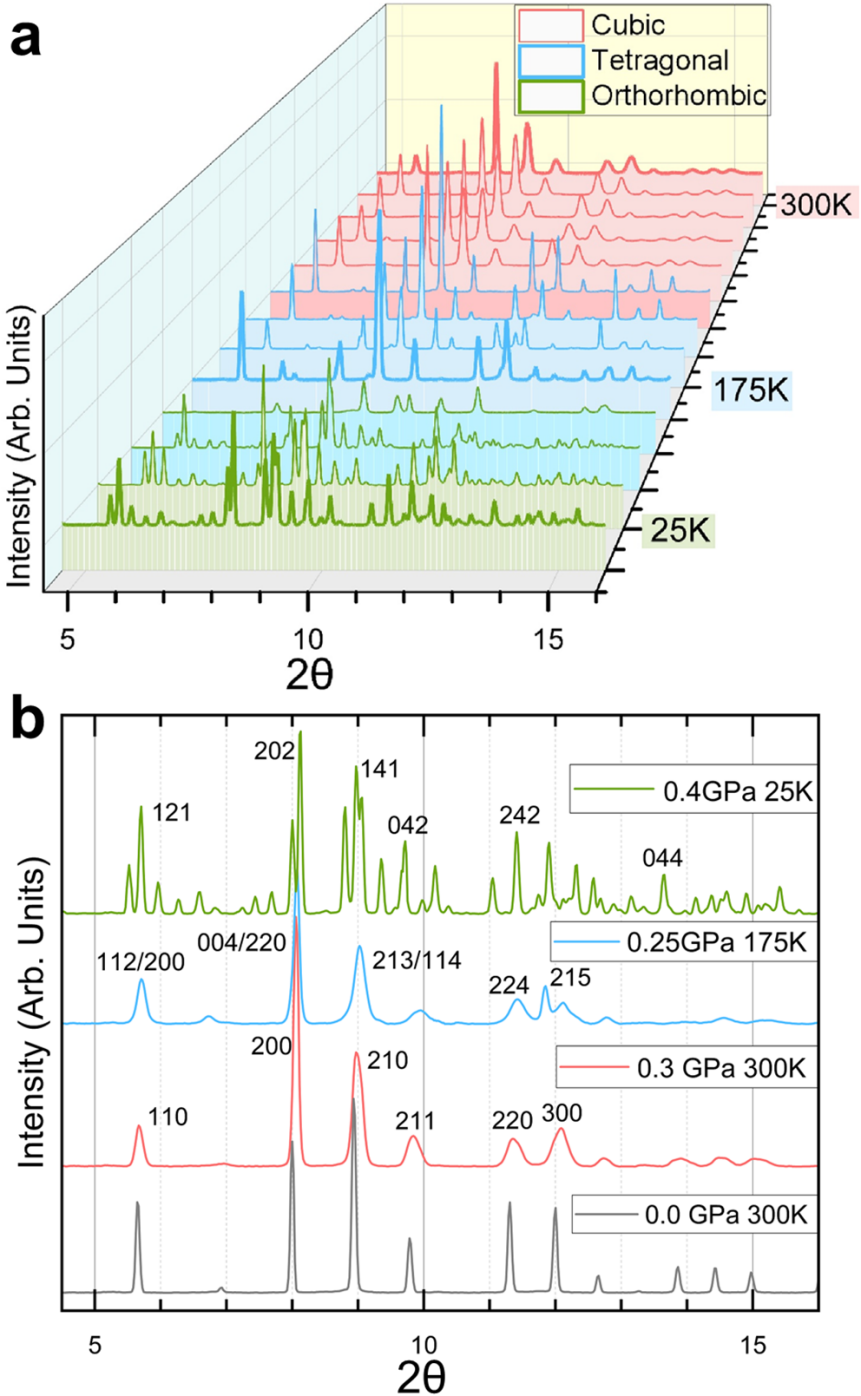
(a) XRD data collected along an approximate isobar showing the evolution of Bragg peaks across two phase transitions. Mixed phase regions are denoted by traces with different colored shading beneath the data. (b) Representative XRD results, corresponding to the shaded boxes in panel (a), from the cubic, tetragonal, and orthorhombic phases of MAPbBr_3_ with Miller indices of high-intensity Bragg peaks indicated for each trace. Initial XRD data at ambient pressure are shown in gray. The sample was then pressurized and cooled to 0.4 GPa and 25K (green) and then warmed back through the tetragonal phase (blue) to the cubic phase (red). The peak breadths clearly increase with compression.

**Figure 2:**
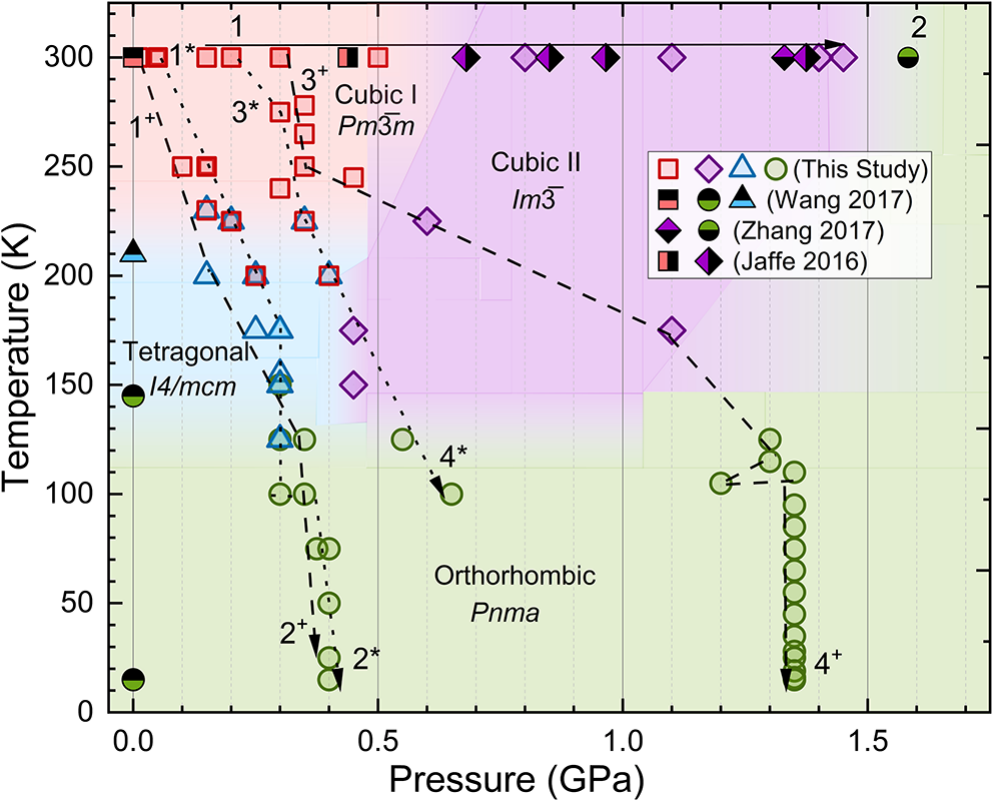
Pressure–temperature phase diagram of MAPbBr_3_ determined in this study, with comparison to previously reported data. Numbered dashed lines represent the experimental pathways followed during measurements; superscripts * and + denote data collected using different DACs. Red, square markers are used for the cubic *Pm*3̅*m* phase, purple diamonds for the cubic *Im*3̅ phase, blue triangles for the tetragonal phase, and green diamonds for the orthorhombic phase. Symbols for our data containing dots or crosses denote the separate experimental pathways. Previously reported data are indicated by half-shaded symbols. The single-phase regions indicated by the shaded background are approximations based on our data and do not necessarily reflect the accurate phase boundaries.

Figure 2 extends the known (*P, T*) phase space to 1.3 GPa at 15 K by using direct crystallographic evidence. Two known pressure-induced structural phase transitions for MAPbBr_3_ are observed at ambient temperature: a transition from the cubic *Pm*3̅*m* phase to the intermediate *Im*3̅ phase near 0.6 GPa, followed by a transition to an orthorhombic *Pnma* phase around 0.8 GPa. The associated transition temperatures exhibit a strong pressure dependence that stabilizes the ambient, low-temperature orthorhombic phase at increasingly higher pressures and temperatures. Figure 2 also corroborates our results with previous high-pressure XRD studies at room temperature and low-temperature XRD studies at ambient pressure. Our results, however, contrast with previous studies (not shown) that inferred phase boundaries from dielectric measurements and infrared vibrational modes.^37,38^ In general, we observe that the structural phase transitions all occur at pressures lower than those reported in the spectroscopic studies. These results provide a comprehensive (P, T) phase diagram for MAPbBr_3_ and confirm that applied pressure favors the stabilization of the low-symmetry orthorhombic phase.

The pressure dependence of the unit cell volume, with and without a pressure-transmitting medium (PTM), is used to determine the bulk modulus of the MAPbBr_3_ structure. Figure 3 compares our XRD measurements, conducted with LiF as the PTM, to He-loaded SCXRD and powder XRD data from Zhang et al. and Jaffe et al., respectively.^53,54^ Our measurements with LiF reveal a significantly sharper volume reduction with increasing pressure, with a total reduction of 7–8% up to 0.6 GPa, corresponding to a modulus of 9.3 GPa. In contrast, measurements utilizing He as the PTM exhibit a more gradual compression with only a 3–4% volume reduction over the same pressure range and a bulk modulus of 12.2 GPa.

**Figure 3:**
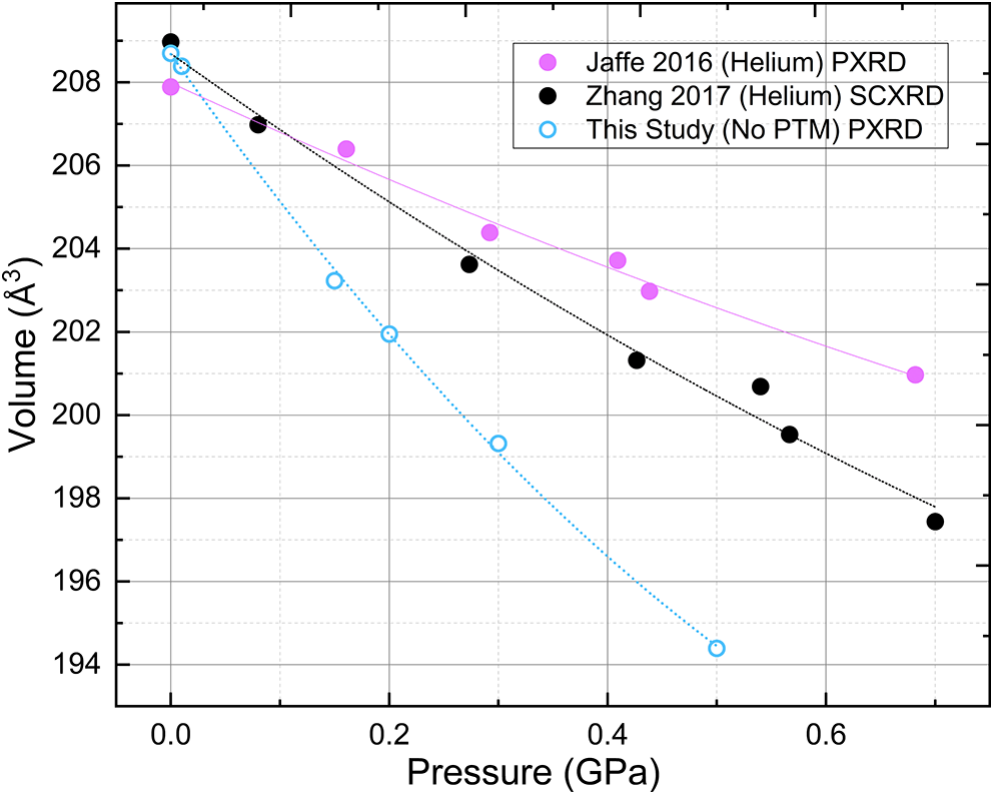
Pressure dependence of the cubic-phase MAPbBr_3_ unit cell. Each data set is fit to a second-order Birch–Murnaghan equation of state to obtain the bulk modulus. Dashed lines illustrate the equation-of-state fits.

### Local Structure from X-ray Absorption Spectroscopy

The pressure and temperature dependence of the local structure in MAPbBr_3_ was investigated by using the extended X-ray absorption fine structure (EXAFS) technique at the Pb L_III_ edge in transmission mode. X-ray absorption was measured radially through the same DAC used for XRD measurements so that the X-rays pass through the Be gasket and LiF PTM, avoiding strong absorption glitches from Bragg peaks of the diamond anvils. The DAC was rotated 90° to switch between the XRD and XAS geometries. Two different cells were used, labeled DAC1 and DAC2. Because of the small beam size (5 × 5 μm^2^) and the DAC geometry, the transmitted flux was low, resulting in a poor signal-to-noise ratio. Consequently, many scans were collected, typically 10–12 for averaging. The scans were averaged in groups of 3 or 4 to have three independent files to check reproducibility and for averaging the fit parameters. However, for DAC1, fewer scans were collected, and, for example, only 6 good scans are available for the sample at 100 K and *P* = 0.7 GPa. Although all scans were centered around the same location, small adjustments were occasionally necessary due to diamond glitches entering the measurement window. These shifts were typically on the order of 8–10 μm, larger than the beam size. This setup suggests that each measurement may have probed a slightly different region of the sample, which could explain why two of the data points exhibit a significantly higher disorder. The *k*-space data are presented in the Supporting Information; these data were Fourier transformed into *r*-space and an example of the *r*-space data is shown as an inset in Figure 4a.

**Figure 4:**
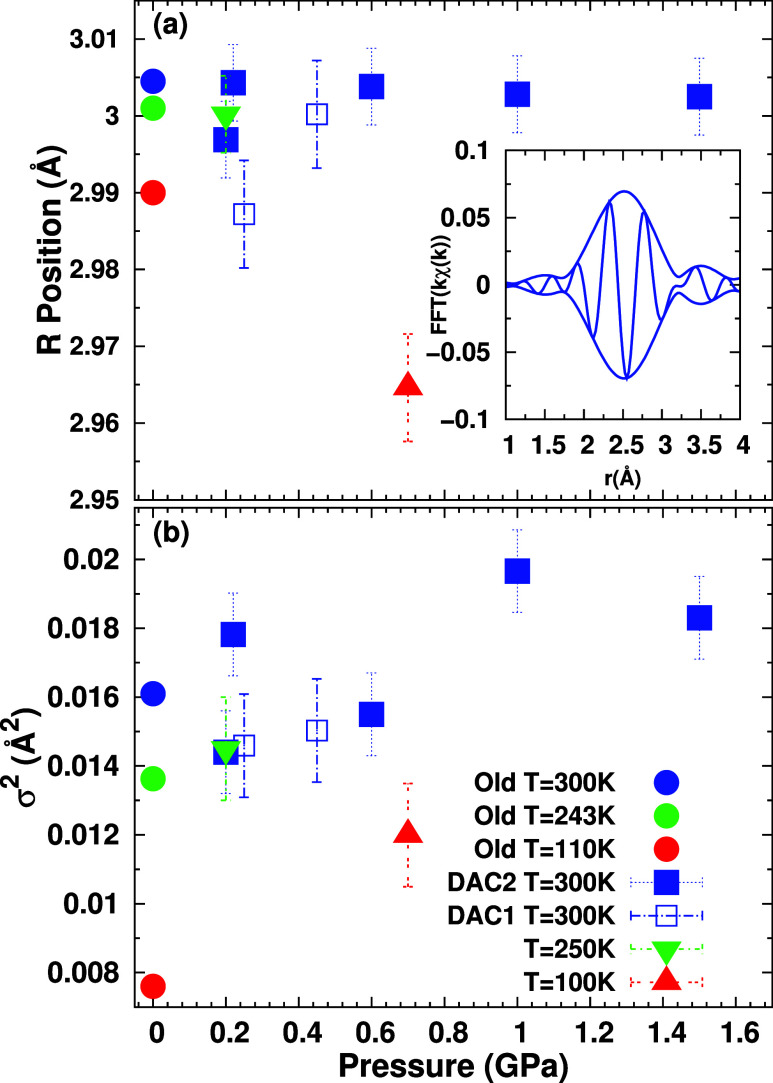
(a) Pb–Br nearest neighbor distance *R* as determined from EXAFS fits. 300 K data from two separately prepared DACs are plotted with shaded and open blue squares (DAC1 and DAC2, respectively). Data from the lower temperature measurements in DAC2 are plotted with green and red triangles. Error bars of 0.005 Å represent the range of *R* obtained when data reduction parameters and FT ranges are varied for DAC2; variations for DAC1 are somewhat larger. Systematic errors may be larger than 0.01 Å. The inset shows a representative Pb–Br PDF generated from a FFT of the *k*-space data. (b) σ^2^ for the Pb–Br bond as a function of pressure from fits of the EXAFS data. Circles are data selected from Weadock et al. at ambient pressure and comparable temperatures to those in the current high-pressure experiments, following the same color scheme as the data from this study.^11^

The average nearest neighbor Pb–Br bond distance, *R*, and mean-squared relative displacement, σ^2^, are calculated from fits of the data using the EXAFS equation (see Methods section) and theoretical functions for the Pb–Br peak calculated using FEFF7.^51^ Because *R* is sensitive to the value of the asymmetry parameter *C*_3_, and there is considerable noise in these data, *C*_3_ values were fixed to the values reported previously for ambient pressure;^11^ the parameters *R* and σ^2^ are plotted in Figure 4a,b, respectively. Our results show good agreement with previous ambient pressure results.^11^ At 300 K, *R* decreases slightly with increasing pressure up to 1.5 GPa while σ^2^ increases about 20% with increasing pressure. Reducing the temperature decreases both *R* and σ^2^.

## Discussion

### Local Structural Changes within PbBr_6_ Octahedra

The global structural changes with pressure and temperature are resolved by XRD. Local structural changes, however, like Jahn–Teller distortions, Pb off-centering (*emphanisis*) or decahedron formation, manifest only in the EXAFS because they are averaged out in the structural refinement.^11,55–59^ Our EXAFS measurements are sensitive to only local structural changes within the PbBr_6_ substructure. The largest impact we observe is an increase in σ^2^ with pressure. This increase is likely a result of a pressure-induced distortion of individual octahedra, manifesting as small, inhomogeneous changes in Pb–Br bond lengths or Br–Pb–Br intraoctahedral bond angles. Homogeneous compression of the PbBr_6_ octahedra with pressure would lead to a reduction in *R*, rather than an increase in σ^2^. Only a very small decrease in *R* is observed in Figure 4a, indicating that homogeneous compression of the octahedra is minimal. We check this result by calculating the bulk modulus *B* of a single PbBr_6_ octahedron to be 32.2 GPa, as derived in SI.

This modulus is much larger than *B* determined from Birch–Murnaghan equation-of-state fits to *V*(*P*) data obtained from XRD. Therefore, we do not expect any uniform compression of the PbBr_6_ octahedra. If we instead consider the effective spring constant of Br motion perpendicular to the Pb–Br bond direction of 0.11 eV/Å^2^,^11^ *B* is reduced to 1.94 GPa, indicating that the octahedra are more susceptible to shear-type deformations than uniform compression or dilation. This *B*_⊥_ is on the order of the pressures used in our experiments, therefore we conclude that the increase in σ^2^ corresponds to a smearing of the Pb–Br bond length by ≈ 0.07 Å from atomic displacements of Pb or Br, in directions perpendicular to the bond.

Alternatively, a pressure-induced Jahn–Teller distortion of the octahedra could increase σ^2^ if the difference between the two Pb–Br bond distances is smaller than the minimum resolvable splitting in our analysis of 0.16 Å. This distortion would also increase the asymmetry of the Pb–Br bond length distribution, captured as the *C*_3_ parameter; however, our data are not of sufficient quality to quantify this. We note that analysis of high-pressure X-ray total scattering measurements of CsPbBr_3_ also concluded that the PbBr_6_ octahedra are deformed under pressure.^40^

## Helium Insertion

Empirical structure maps, often inferred or developed for inorganic perovskites, are often used to predict the phase stability in hybrid organic inorganic perovskites. These maps use parameters like the Goldschmidt tolerance factor and octahedral factor to estimate whether a given ionic combination will favor a high-symmetry cubic structure or a distorted, lower-symmetry phase.^60^ Hybrid perovskites introduce unique challenges particularly due to the organic cation that combines dynamic movement with dipolar or quadrupolar electrostatics and responds differently to applied pressure than the inorganic framework.^28–30,41,44^ This often leads to a complex phase behavior under compression. Indeed, it has been proposed that increasing pressure could suppress dynamic octahedral tilting in a manner analogous to cooling, thereby stabilizing lower-symmetry phases at ambient temperatures.^61^ However, this idea has been challenged by contradictory results in oxide perovskites, where compressibility differences between the octahedra and the A-site framework lead to diverse and sometimes unexpected pressure responses.^62^

In this work, we investigate the insertion of an inert species, specifically He, as a means to stabilize the cubic phase without altering the chemical or electronic landscape of the material. Experimental evidence supports the plausibility of this approach. Figure 3 shows that the MAPbBr_3_ unit cell volume decreases by only 3–4% with a He PTM whereas the decrease is 7–8% with an LiF PTM. We hypothesize that He inserts into the cuboctahedral spaces where the MA^+^ cation resides. “Filling” this space with an inert atom then provides mechanical resistance to octahedral tilting and structural deformations, increasing the bulk modulus. There are no reports indicating that the unit cell volume increases for MAPbBr_3_ samples compressed in He, in contrast to the behavior observed for CaZrF_6_, CaNbF_6_, and ScF_3_.^54,63^ The Pb–Pb distance in MAPbBr_3_ is 5.93 Å, significantly larger than the corresponding distances of 4.04 Å, 4.24 Å, and 4.20 Å in ScF_3_, CaZrF_6_, and CaNbF_6_, respectively.^63^ This disparity suggests that the spaces that He occupies in MAPbBr_3_ are larger than in the other systems, and therefore, He insertion produces no observable effects on the unit cell volume.

Our findings are consistent with earlier high-pressure studies. Zheng et al. reported that MAPbBr_3_ maintained cubic symmetry under pressure in the presence of He, with no signs of amorphization.^54^ Jaffe et al., similarly observed persistent Bragg peaks despite the appearance of a diffuse scattering halo, again suggesting structural coherence in a pseudocubic form.^53^ However, in both studies, parameters such as the rate of pressurization and duration of He exposure, were not carefully controlled. These factors could significantly affect the extent of He diffusion into the lattice. Notably, the smallest compressibility is observed in powder samples that we expect to have the largest He uptake due to a large surface area and small particle size.

We investigate this He-mediated stabilization hypothesis mechanism below. Density functional theory is used to calculate the thermodynamics of forming MAPbBr_3_He_*x*_ (*x* = 0.5 and 1) under pressures ranging from 1 to 3 GPa. Figure 5 plots the difference in calculated Gibbs free energies (Δ*G*) between He-containing structures and their separate components (MAPbBr_3_ + He) as a function of temperature. We see that the Δ*G* values are on the order of 0–10 meV/atom, indicating that such incorporation is energetically accessible. The thermal energy at ambient temperature (*k*_B_ *T* ≈ 25.7 meV) is greater than the calculated Δ*G*, suggesting that a pressure-stabilized, He-containing structure could form at temperatures that both overcome the free energy barrier for insertion and provide an adequate diffusion coefficient of He into the structure. Moreover, lower molar fractions of He (*x* < 1) are more thermodynamically favorable, particularly at higher pressures and lower temperatures. The less positive Δ*G* at higher pressures is likely due to an increase in Δ*S*; the high compressibility of He suggests that the molar volume of He in the perovskite structure is greater than the molar volume in the condensed phase. These calculations point to partial He incorporation as a realistic and effective route for stabilizing the structure of open framework and hybrid materials.

**Figure 5:**
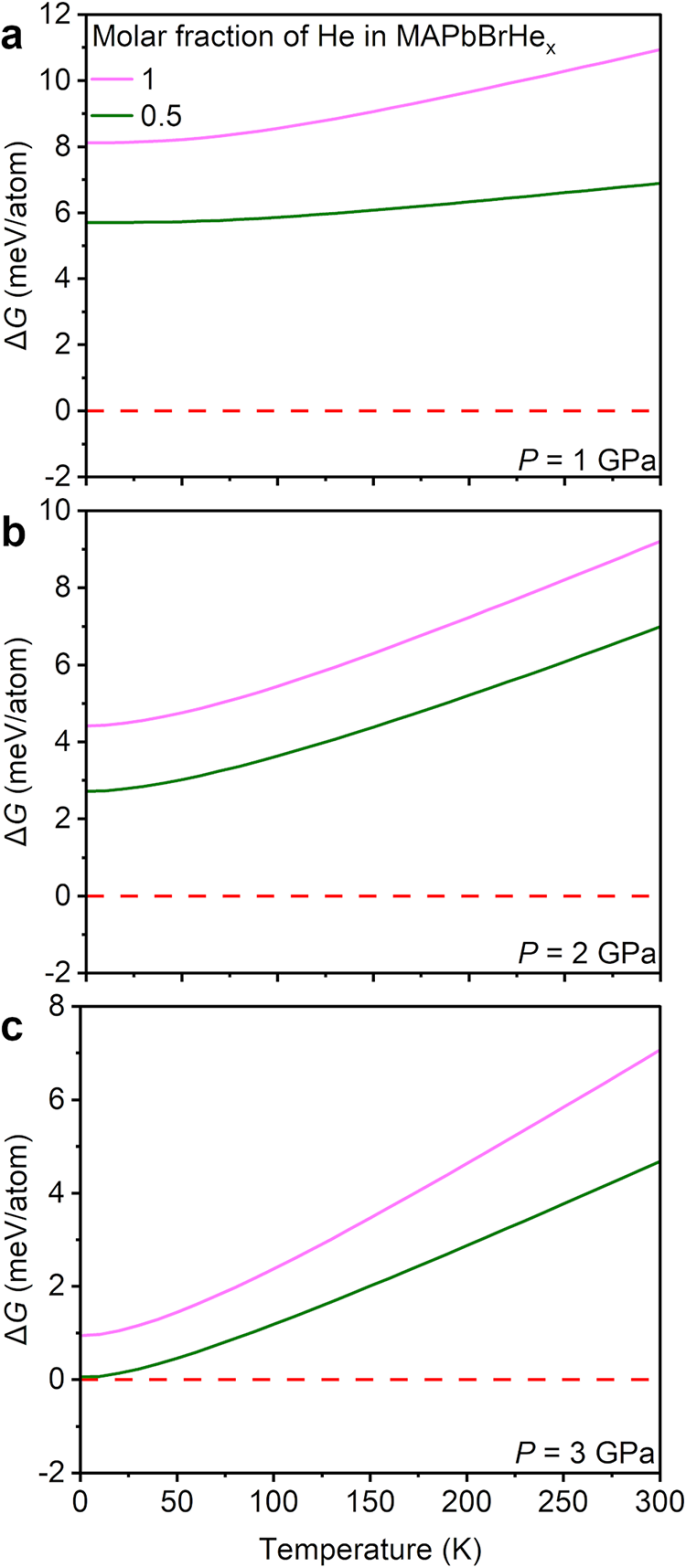
Gibbs free energy changes, Δ*G* = Δ*H* – *T*Δ*S*, as a function of the temperature and molar fraction of He insertion into MAPbBr_3_ at various pressures (*k*_*B*_ *T* ≈ 25.7 meV at 298 K).

The structural changes associated with He insertion are investigated with *ab initio* molecular dynamics (AIMD) simulations. The NPT ensemble is used to simulate the MAPbBr_3_ and MAPbBr_3_He structures at 0.3 GPa and 300 K. Figure 6a shows the 0 K DFT-optimized structure. X-ray diffraction patterns are calculated from the 0 K DFT structures and the 300 K AIMD average structures of MAPbBr_3_ and MAPbBr_3_He at 0.3 GPa and are compared in Figure 6b,c. At both temperatures, He resides between the MA^+^-occupied cuboctahedral interstices on pseudocubic {110} planes. Global structural changes are inferred from the calculated diffraction patterns in Figure 6b,c. The pure MAPbBr_3_ compound shows subtle changes in the structure between 0 and 300 K at 0.3 GPa. In Figure 6c, however, it is clear that He insertion favors a higher-symmetry structure at 300 K (fewer Bragg peaks) compared to both the 0 K structure and the 300 K He-free structure. This is a pseudocubic phase that remains structurally coherent under conditions where the pristine material may undergo a symmetry-lowering transition.

**Figure 6:**
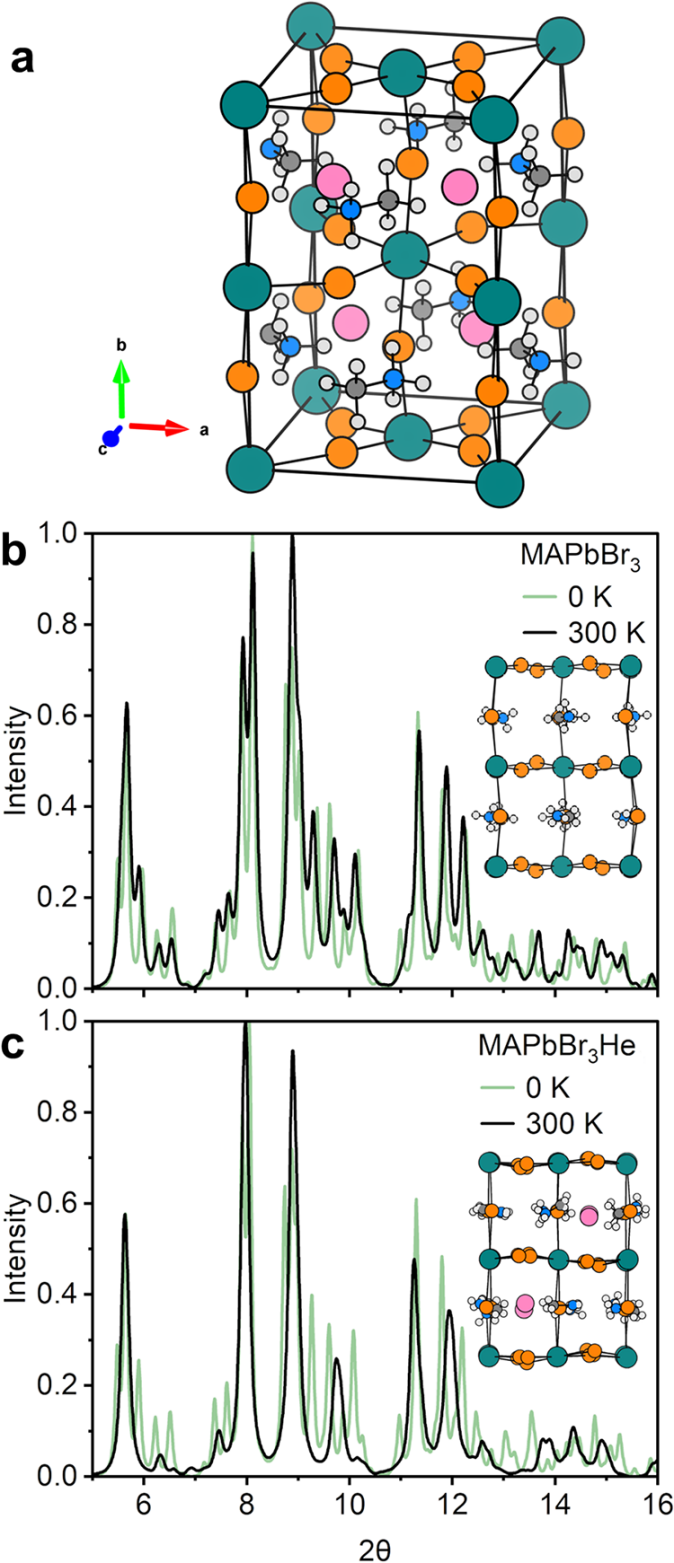
(a) DFT-optimized structure of MAPbBr_3_He at 1 GPa. Teal atoms are Pb, orange Br, blue N, dark gray C, light gray H, and pink are He. Powder X-ray diffraction patterns (λ = 0.4133 Å) calculated from the time-averaged structure of (b) MAPbBr_3_ and (c) MAPbBr_3_He_1_ over the AIMD simulations at 300 K and 0.3 GPa (black), compared to the calculated diffraction pattern of the same structures optimized with DFT at 0 K (green).

Helium insertion into MAPbBr_3_ offers a promising pathway for stabilizing the cubic phase and enhancing the structural robustness under pressure. This robustness may be maintained at ambient conditions if He does not leave the perovskite when sealed in a device. Unlike ionic substitution, this approach decouples structure stabilization from changes in electronic properties, providing a valuable strategy for improving the performance and durability of hybrid perovskites in photovoltaic and optoelectronic applications. More broadly, these findings highlight the importance of pressure-transmitting environments in high-pressure studies and suggest that inert gas insertion may be a general tool for tuning phase stability in soft, deformable crystal systems.

## Conclusions

In this study, we present a comprehensive pressure–temperature phase diagram of MAPbBr_3_, combining *in situ* synchrotron X-ray diffraction and extended X-ray absorption fine structure measurements. Our results extend the known structural phase space of MAPbBr_3_ to higher pressures (up to 1.5 GPa) and lower temperatures (down to 15 K) based on crystallographic data, confirming that compression stabilizes the orthorhombic phase and suppresses higher-symmetry phases.

EXAFS analysis provided insight into the pressure dependence of the local structural disorder. We observed a monotonic increase in σ^2^ with pressure, suggesting PbBr_6_ octahedra distort rather than undergo homogeneous compression. This disorder is distinct from the lower-symmetry dynamic local order observed in LHPs at ambient pressure and warrants further study of the subsequent effects on optoelectronic properties.

Finally, a notable outcome of this work is the experimental and theoretical evidence of helium diffusion and intercalation into the MAPbBr_3_ structure when used as a pressure-transmitting medium. We show that helium incorporation leads to a measurable reduction in compressibility and stabilizes a pseudocubic structure under pressure. Density functional theory and AIMD simulations predict that helium atoms can occupy interstitial sites with minimal energetic cost, suppressing octahedral tilting. We propose inert atom intercalation as a pathway to enhance structural stability in hybrid perovskites that circumvents the optoelectronic changes associated with tailoring the Goldschmidt tolerance factor. This insight may be extended to broader classes of hybrid materials and offers promising strategies for the development of robust materials for photovoltaic and optoelectronic applications.

## Supplementary Material





## Notes

Computational crystal structure data (ZIP)
Powder XRD data, a schematic of the experimental setup for synchrotron XRD and XAS measurements within a DAC, images of DFT-optimized structures, additional EXAFS data, and the derivation of bulk modulus for a single PbBr_6_ octahedra in a corner-sharing, simple-cubic configuration (PDF)

## References

[1] Green M. A., Dunlop E. D., Yoshita M., Kopidakis N., Bothe K., Siefer G., Hao X., Jiang J. Y. (2025). Solar Cell
Efficiency Tables (Version 66). Prog. Photovoltaics:
Res. Appl..

[2] Egger D. A., Bera A., Cahen D., Hodes G., Kirchartz T., Kronik L., Lovrincic R., Rappe A. M., Reichman D. R., Yaffe O. (2018). What Remains Unexplained
about the Properties of Halide Perovskites?. Adv. Mater..

[3] Liu Q., Ren H., Wei Q., Li M. (2025). Multifunctional Chiral Halide Perovskites:
Advancing Chiro-Optics, Chiro-Optoelectronics, and Spintronics. Adv. Sci..

[4] Zhang C., Ono L. K., Qi Y. (2024). Color/Spectral
Stability of Mixed
Halide Perovskite Light-Emitting Diodes. Adv.
Funct. Mater..

[5] Wei H., Huang J. (2019). Halide lead perovskites
for ionizing radiation detection. Nat. Commun..

[6] Chen M., Dong Y., Zhang Y. (2024). Stress Engineering for
Mitigating Thermal Cycling Fatigue in Perovskite Photovoltaics. ACS Energy Lett..

[7] McAndrews G. R., Guo B., Morales D. A., Amassian A., McGehee M. D. (2023). How the dynamics
of attachment to the substrate influence stress in metal halide perovskites. APL Energy.

[8] Schelhas L. T., Christians J. A., Berry J. J., Toney M. F., Tassone C. J., Luther J. M., Stone K. H. (2016). Monitoring a Silent Phase Transition
in CH_3_NH_3_PbI_3_ Solar Cells via Operando
X-ray Diffraction. ACS Energy Lett..

[9] Mundt L. E., Zhang F., Palmstrom A. F., Xu J., Tirawat R., Kelly L. L., Stone K. H., Zhu K., Berry J. J., Toney M. F., Schelhas L. T. (2022). Mixing Matters:
Nanoscale Heterogeneity
and Stability in Metal Halide Perovskite Solar Cells. ACS Energy Lett..

[10] Mundt L. E., Schelhas L. T. (2020). Structural Evolution During Perovskite
Crystal Formation
and Degradation: In Situ and Operando X-Ray Diffraction Studies. Adv. Energy Mater..

[11] Weadock N. J., MacKeen C., Qin X., Waquier L., Rakita Y., Vigil J. A., Karunadasa H. I., Blum V., Toney M. F., Bridges F. (2023). Thermal Contributions
to the Local and Long-Range Structural
Disorder in CH_3_NH_3_PbBr_3_. PRX Energy.

[12] Prasanna R., Gold-Parker A., Leijtens T., Conings B., Babayigit A., Boyen H.-G., Toney M. F., McGehee M. D. (2017). Band Gap
Tuning
via Lattice Contraction and Octahedral Tilting in Perovskite Materials
for Photovoltaics. J. Am. Chem. Soc..

[13] Li Z., Yang M., Park J.-S., Wei S.-H., Berry J. J., Zhu K. (2016). Stabilizing Perovskite
Structures by Tuning Tolerance Factor: Formation
of Formamidinium and Cesium Lead Iodide Solid-State Alloys. Chem. Mater..

[14] Barrier J., Beal R. E., Gold-Parker A., Vigil J. A., Wolf E., Waquier L., Weadock N. J., Zhang Z., Schelhas L. T., Nogueira A. F., McGehee M. D., Toney M. F. (2021). Compositional heterogeneity
in Cs_(y)_FA_(1-y)_Pb­(Br_[x]_I_[1-x]_)_3_ perovskite films and its impact on phase behavior. Energy Environ. Sci..

[15] Šimėnas M., Balčiu̅nas S., Svirskas u., Kinka M., Ptak M., Kalendra V., Ga̧gor A., Szewczyk D., Sieradzki A., Grigalaitis R., Walsh A., Ma̧czka M., Banys J. (2021). Phase Diagram and Cation
Dynamics of Mixed MA_1-x_FA_x_PbBr_3_ Hybrid
Perovskites. Chem. Mater..

[16] Simenas M., Balciunas S., Wilson J. N., Svirskas S., Kinka M., Garbaras A., Kalendra V., Gagor A., Szewczyk D., Sieradzki A., Maczka M., Samulionis V., Walsh A., Grigalaitis R., Banys J. (2020). Suppression of phase
transitions and glass phase signatures in mixed cation halide perovskites. Nat. Commun..

[17] Schwarz U., Hillebrecht H., Kaupp M., Syassen K., von Schnering H. G., Thiele G. (1995). Pressure-Induced Phase Transition in CsGeBr_3_ Studied by X-Ray Diffraction and Raman Spectroscopy. J. Solid State Chem..

[18] Seo D.-K., Gupta N., Whangbo M.-H., Hillebrecht H., Thiele G. (1998). Pressure-Induced Changes in the Structure and Band
Gap of CsGeX_3_ (X = Cl, Br) Studied by Electronic Band Structure
Calculations. Inorg. Chem..

[19] Liang A., Gonzalez-Platas J., Turnbull R., Popescu C., Fernandez-Guillen I., Abargues R., Boix P. P., Shi L.-T., Errandonea D. (2022). Reassigning
the Pressure-Induced Phase Transitions of Methylammonium Lead Bromide
Perovskite. J. Am. Chem. Soc..

[20] Boström H. L.
B., Goodwin A. L. (2021). Hybrid
Perovskites, Metal-Organic Frameworks, and Beyond:
Unconventional Degrees of Freedom in Molecular Frameworks. Acc. Chem. Res..

[21] Hutter E. M., Muscarella L. A., Wittmann F., Versluis J., McGovern L., Bakker H. J., Woo Y.-W., Jung Y.-K., Walsh A., Ehrler B. (2020). Thermodynamic Stabilization of Mixed-Halide
Perovskites
against Phase Segregation. Cell Rep. Phys. Sci..

[22] McGovern L., Grimaldi G., Futscher M. H., Hutter E. M., Muscarella L. A., Schmidt M. C., Ehrler B. (2021). Reduced Barrier for Ion Migration
in Mixed-Halide Perovskites. ACS Appl. Energy
Mater..

[23] Muscarella L. A., Hutter E. M., Wittmann F., Woo Y. W., Jung Y.-K., McGovern L., Versluis J., Walsh A., Bakker H. J., Ehrler B. (2020). Lattice Compression Increases the Activation Barrier
for Phase Segregation in Mixed-Halide Perovskites. ACS Energy Lett..

[24] Wang Y., Lü X., Yang W., Wen T., Yang L., Ren X., Wang L., Lin Z., Zhao Y. (2015). Pressure-Induced Phase
Transformation, Reversible Amorphization, and Anomalous Visible Light
Response in Organolead Bromide Perovskite. J.
Am. Chem. Soc..

[25] Francisco-López A., Charles B., Weber O. J., Alonso M. I., Garriga M., Campoy-Quiles M., Weller M. T., Goñi A. R. (2018). Pressure-Induced
Locking of Methylammonium Cations versus Amorphization in Hybrid Lead
Iodide Perovskites. J. Phys. Chem. C.

[26] Capitani F., Marini C., Caramazza S., Dore P., Pisanu A., Malavasi L., Nataf L., Baudelet F., Brubach J.-B., Roy P., Postorino P. (2017). Locking of
Methylammonium by Pressure-Enhanced H-Bonding
in (CH_3_NH_3_)­PbBr_3_ Hybrid Perovskite. J. Phys. Chem. C.

[27] Liang A., Turnbull R., Popescu C., Fernandez-Guillen I., Abargues R., Boix P. P., Errandonea D. (2023). Pressure-Induced
Phase Transition versus Amorphization in Hybrid Methylammonium Lead
Bromide Perovskite. J. Phys. Chem. C.

[28] Li J., Wang Y., Saha S., Chen Z., Hofmann J., Misleh J., Chapman K. W., Reimer J. A., Filip M. R., Karunadasa H. I. (2024). 3D Lead-Organoselenide-Halide
Perovskites and their
Mixed-Chalcogenide and Mixed-Halide Alloys. Angew. Chem., Int. Ed..

[29] Li J., Chen Z., Saha S., Utterback J. K., Aubrey M. L., Yuan R., Weaver H. L., Ginsberg N. S., Chapman K. W., Filip M. R., Karunadasa H. I. (2022). Zwitterions
in 3D Perovskites: Organosulfide-Halide Perovskites. J. Am. Chem. Soc..

[30] Li J., Hofmann J., Stolz R. M., Wen J., Deschene C. R., Bartels H., Liu Z., Salleo A., Lin Y., Chapman K. W., Karunadasa H. I. (2025). Suppressing Phase Transitions and
High-Pressure Amorphization through Tethered Organic Cations in Organochalcogenide-Halide
Perovskites. J. Am. Chem. Soc..

[31] Dubajic M., Neilson J. R., Klarbring J. (2025). Dynamic nanodomains
dictate macroscopic properties in lead halide perovskites. Nat. Nanotechnol..

[32] Baldwin W. J., Liang X., Klarbring J., Dubajic M., Dell’Angelo D., Sutton C., Caddeo C., Stranks S. D., Mattoni A., Walsh A., Csányi G. (2024). Dynamic Local
Structure in Caesium
Lead Iodide: Spatial Correlation and Transient Domains. Small.

[33] Lanigan-Atkins T., He X., Krogstad M. J., Pajerowski D. M., Abernathy D. L., Xu G. N. M. N., Xu Z., Chung D.-Y., Kanatzidis M. G., Rosenkranz S., Osborn R., Delaire O. (2021). Two-dimensional overdamped
fluctuations of the soft perovskite lattice in CsPbBr_3_. Nat. Mater..

[34] Weadock N. J., Sterling T. C., Vigil J. A. (2023). The nature of dynamic
local order in CH_3_NH_3_PbI_3_ and CH_3_NH_3_PbBr_3_. Joule.

[35] Weadock N. J., Gehring P. M., Gold-Parker A., Smith I. C., Karunadasa H. I., Toney M. F. (2020). Test of the Dynamic-Domain
and Critical Scattering
Hypotheses in Cubic Methylammonium Lead Triiodide. Phys. Rev. Lett..

[36] Bridges F., Gruzdas J., MacKeen C., Mayford K., Weadock N. J., Baltazar V. U., Rakita Y., Waquier L., Vigil J. A., Karunadasa H. I., Toney M. F. (2023). Local structure, bonding, and asymmetry
of ((NH_2_)_2_CH)­PbBr_3_, CsPbBr_3_, and (CH_3_NH_3_)­PbBr_3_. Phys. Rev. B.

[37] Chan Y. T., Elliger N., Klis B., Kollár M., Horváth E., Forró L., Dressel M., Uykur E. (2022). High-pressure
investigations in CH_3_NH_3_PbX_3_ (X =
I, Br, and Cl): Suppression of ion migration and stabilization of
low-temperature structure. Phys. Rev. B.

[38] Gesi K. (1997). Effect of
hydrostatic pressure on the structural phase transitions in CH_3_NH_3_PbX_3_ (X = Cl, Br, I). Ferroelectrics.

[39] Marin-Villa P., Gaboardi M., Joseph B., Alabarse F., Armstrong J., Drużbicki K., Fernandez-Alonso F. (2025). Methylammonium
Lead Iodide across
Physical Space: Phase Boundaries and Structural Collapse. J. Phys. Chem. Lett..

[40] Celeste A., Girdzis S. P., Cladek B. R., Deschene C. R., Wolf N. R., Chapman K. W., Karunadasa H. I., Tucker M. G., Mao W. L., Lin Y. (2025). Total X-ray scattering
and big-box modeling of pressure-induced local
disorder and partial amorphization in CsPbBr3. Nat. Commun..

[41] Racioppi S., Miao M., Zurek E. (2023). Intercalating
Helium into A-Site
Vacant Perovskites. Chem. Mater..

[42] Hester B. R., dos Santos A. M., Molaison J. J., Hancock J. C., Wilkinson A. P. (2017). Synthesis
of Defect Perovskites (He_2-x_ □_x_)­(CaZr)­F_6_ by Inserting Helium into the Negative Thermal Expansion Material
CaZrF6. J. Am. Chem. Soc..

[43] Lloyd A. J. I., Hester B. R., Baxter S. J., Ma S., Prakapenka V. B., Tkachev S. N., Park C., Wilkinson A. P. (2021). Hybrid
Double Perovskite Containing Helium: [He_2_]­[CaZr]­F_6_. Chem. Mater..

[44] Ma S., Hester B. R., Lloyd A. J. I., dos Santos A. M., Molaison J. J., Wilkinson A. P. (2024). Synthesis
and Properties of the Helium
Clathrate and Defect Perovskite [He_2-x_ □_x_]­[CaNb]­F_6_. J. Phys. Chem. C.

[45] Sato T., Funamori N., Yagi T. (2011). Helium penetrates
into silica glass
and reduces its compressibility. Nat. Commun..

[46] Londono D., Finney J. L., Kuhs W. F. (1992). Formation, stability, and structure
of helium hydrate at high pressure. J. Chem.
Phys..

[47] Guńka P. A., Dziubek K. F., Gładysiak A., Dranka M., Piechota J., Hanfland M., Katrusiak A., Zachara J. (2015). Compressed Arsenolite
As_4_O_6_ and Its Helium Clathrate As_4_O_6_·2He. Cryst. Growth Des..

[48] Guńka P. A., Hapka M., Hanfland M., Dranka M., Chałasiński G., Zachara J. (2018). How and Why
Does Helium Permeate Nonporous Arsenolite
Under High Pressure?. ChemPhysChem.

[49] Saidaminov, M. I. ; Abdelhady, A. L. ; Murali, B. ; Alarousu, E. ; Burlakov, V. M. ; Peng, W. ; Dursun, I. ; Wang, L. ; He, Y. ; Maculan, G. ; Goriely, A. ; Wu, T. ; Mohammed, O. F. ; Bakr, O. M. High-quality bulk hybrid perovskite single crystals within minutes by inverse temperature crystallization. 6, 7586. 10.1038/ncomms8586 PMC4544059 26145157

[50] Booth, C. H. R-space X-ray absorption package. 2010, p 2010.

[51] Ankudinov A. L., Rehr J. J. (1997). Relativistic calculations
of spin-dependent x-ray-absorption
spectra. Phys. Rev. B.

[52] Blöchl P. E. (1994). Projector
augmented-wave method. Phys. Rev. B.

[53] Jaffe A., Lin Y., Beavers C. M., Voss J., Mao W. L., Karunadasa H. I. (2016). High-Pressure
Single-Crystal Structures of 3D Lead-Halide Hybrid Perovskites and
Pressure Effects on Their Electronic and Optical Properties. ACS Cent. Sci..

[54] Zhang R., Cai W., Bi T., Zarifi N., Terpstra T., Zhang C., Verdeny Z. V., Zurek E., Deemyad S. (2017). Effects of Nonhydrostatic
Stress on Structural and Optoelectronic Properties of Methylammonium
Lead Bromide Perovskite. J. Phys. Chem. Lett..

[55] Laurita G., Fabini D. H., Stoumpos C. C., Kanatzidis M. G., Seshadri R. (2017). Chemical Tuning of Dynamic Cation
Off-Centering in
the Cubic Phases of Hybrid Tin and Lead Halide Perovskites. Chem. Sci..

[56] Fabini D. H., Seshadri R., Kanatzidis M. G. (2020). The Underappreciated Lone Pair in
Halide Perovskites Underpins Their Unusual Properties. MRS Bull..

[57] Yi S., Lee J.-H. (2022). Degenerate Lattice-Instability-Driven Amorphization
under Compression in Metal Halide Perovskite CsPbI3. J. Phys. Chem. Lett..

[58] Kwei G. H., Billinge S. J. L., Cheong S.-W., Saxton J. G. (1995). Pair-Distribution
Functions of Ferroelectric Perovskites: Direct Observation of Structural
Ground States. Ferroelectrics.

[59] Ravel B., Stern E. A., Vedrinskii R. I., Kraizman V. (1998). Local Structure and
the 3. Ferroelectrics.

[60] Bartel C. J., Sutton C., Goldsmith B. R., Ouyang R., Musgrave C. B., Ghiringhelli L. M., Scheffler M. (2019). New tolerance factor to predict the
stability of perovskite oxides and halides. Sci. Adv..

[61] Yang D., Fu Y., Sun Y., Li Y., Wang K., Xiao Z., Biswas K., Zhang L. (2021). Phase transition
pathway of hybrid
halide perovskites under compression: Insights from first-principles
calculations. Phys. Rev. Mater..

[62] Aguado F., Rodríguez F., Valiente R., Itiè J.-P., Hanfland M. (2012). Pressure effects on
Jahn-Teller distortion in perovskites:
The roles of local and bulk compressibilities. Phys. Rev. B.

[63] Ma S., Baxter S. J., Park C., Chariton S., dos Santos A. M., Molaison J. J., Wilkinson A. P. (2025). Helium Incorporation into Scandium
Fluoride, a Model Negative Thermal Expansion Material. Chem. Mater..

