# Supplementary material for: Pressure-Driven Helium Insertion for Structural Stability of CH3NH3PbBr3 Hybrid Perovskites
Source: Chem Mater. 2026 Mar 31;38(7):3439–48. doi: 10.1021/acs.chemmater.5c03238 (PMC13084985; doi:10.1021/acs.chemmater.5c03238)
Supplement: Supplementary file 2 [file cm5c03238_si_002.pdf]

Supporting Information for:Pressure-driven helium insertion for structural stability of $CH_3NH_3PbBr_3$ hybrid perovskites

Nicholas J. Weadock,[*,†] Willis Holle,[‡] Kiley Mayford,[¶] Stefano Racioppi,[§,∥] Anukriti Ghimire,[‡] Dylan Ladd,[†] Changyong Park,[⊥] Eva Zurek,[§] Michael F. Toney,[†,#,@] Frank Bridges,[¶] and Shanti Deemyad[*,‡]

†*Materials Science and Engineering, University of Colorado, Boulder, CO 80309, USA*

‡*Department of Physics and Astronomy, University of Utah, Salt Lake City, UT, 84112, USA*

¶*Physics Department, University of California, Santa Cruz, CA 95064, USA*

§*Department of Chemistry, State University of New York at Buffalo, Buffalo, NY 14260, USA*

∥*Department of Materials Science and Metallurgy, University of Cambridge, Cambridge CB30FS, UK*

⊥*High Pressure Collaborative Access Team (HPCAT), X-ray Science Division, Argonne National Laboratory, Lemont, Illinois 60439, USA*

#*Department of Chemical and Biological Engineering, University of Colorado, Boulder, CO 80309, USA*

@*Renewable and Sustainable Energy Institute (RASEI), University of Colorado, Boulder, CO 80309, USA*

E-mail: nicholas.weadock@colorado.edu; shanti.deemyad@utah.edu

XRD characterization of $MAPbBr_3$ powders

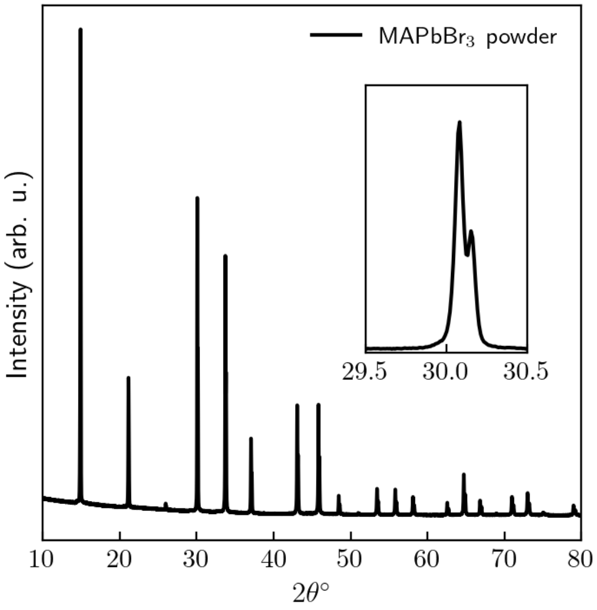

Figure S1: XRD data of the ground and annealed $MAPbBr_3$ powder prepared for diamond anvil cell experiments. Inset shows how the Kα splitting is observable at low 2θ angles, indicating that the grinding and annealing procedure introduces minimal defects or nanocrystallinity.

Diamond anvil cell experimental setup

A schematic illustrating the experimental setup for X-ray diffraction and X-ray absorption spectroscopy measurements in the same diamond anvil cell (DAC) is provided in Figure S2. The $MAPbBr_3$ powders are loaded into the DAC using a Be gasket and LiF pressure transmitting medium (chosen for low X-ray absorption). XRD measurements are taken in transmission mode through the diamonds and data is collected on an area detector. The images on the right-hand side show the powder loaded into the DAC (top), a representative image on the area detector (middle) and an X-ray transmission line scan (XRD orientation) plot showing the $MAPbBr_3$ sample (bottom). Three spots in the dark, low transmission region correspond to representative positions for $MAPbBr_3$ diffraction measurements, and one spot

in the light, high transmission region shows the position for LiF diffraction measurements. XAS measurements are acquired by rotating the DAC 90 degrees and measuring X-ray transmission through the Be gasket using ionization chambers. The X-ray transmission of the $MAPbBr_3$ sample in the radial direction (XAS orientation) is shown in the top right panel, with the dark regions corresponding to $MAPbBr_3$. A representative X-ray absorption spectrum is plotted in the top left.

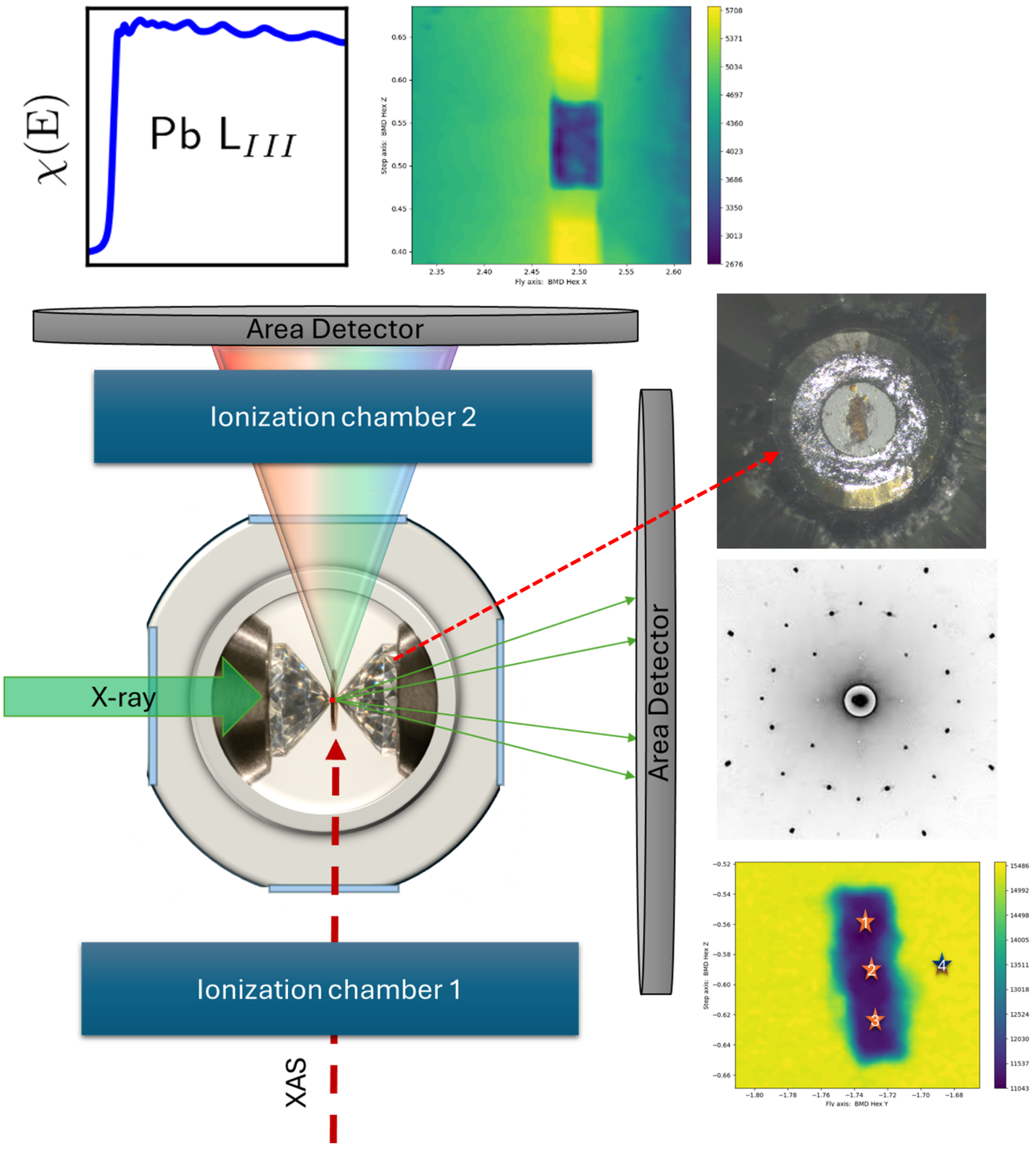

Figure S2: Experimental setup for high pressure measurements.

DFT optimized structures

The conventional unit cells for DFT optimized structures of $MAPbBr_3He_x$, (x = 0, 0.5, 1) are displayed in Figure S3. For x = 0, 1, the orthorhombic *Pnma* structure was found to be dynamically stable whereas for x = 0.5, the lower symmetry monoclinic $P2_1/c$ structure is needed.

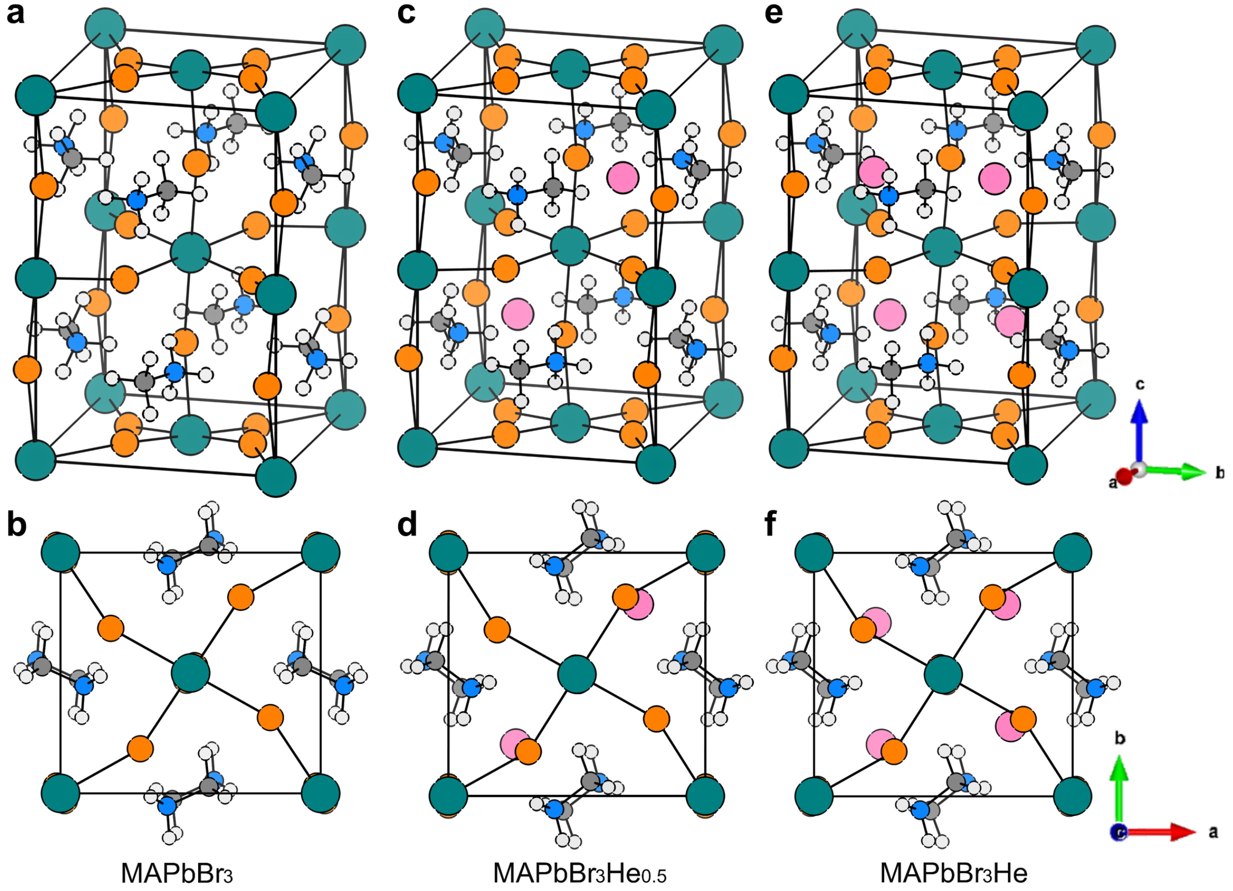

Figure S3: Visualization of the optimized structures for a,b $MAPbBr_3$, c,d $MAPbBr_3He_{0.5}$, and e,f $MAPbBr_3He$. b,d,f are viewed down the c-axis. Pb atoms are blue-green, Br are orange, He are pink, N are blue, C are gray, and H are off-white.

X-ray absorption spectroscopy data

The reduced Pb L_{III} X-ray absorption data, processed according to the Methods section of the Main Text, are produced below in Figure S4. The r-space data obtained from a Fourier transform of the k-space data are shown in Figure S5.

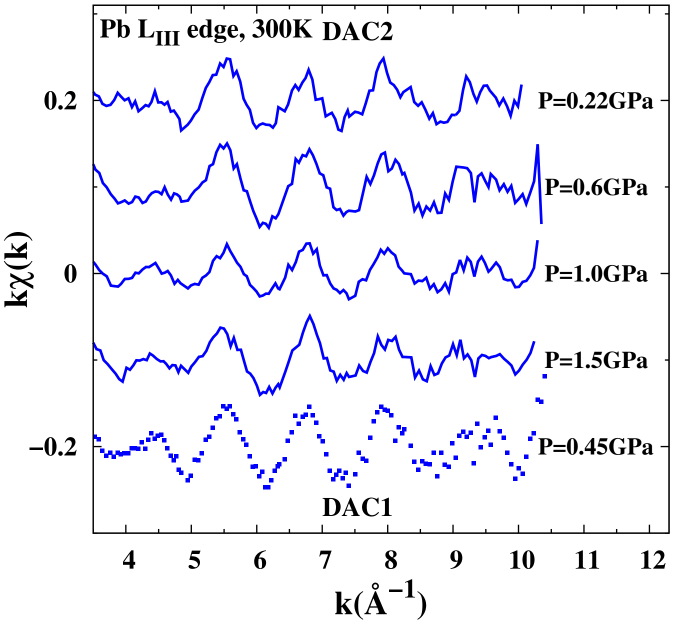

Figure S4: Examples of the k-space data collected in the diamond anvil cells (DAC) at 300K. Data were collected at several pressures up to 1.5 GPa. The plots for DAC2 are an average over 12 scans and have the better signal to noise. One example of the data collected in DAC1 is shown in squares for P = 0.45 GPa; it is an average over 6 scans and the noise is larger. The upturn at the end of each scan is from the Br K edge.

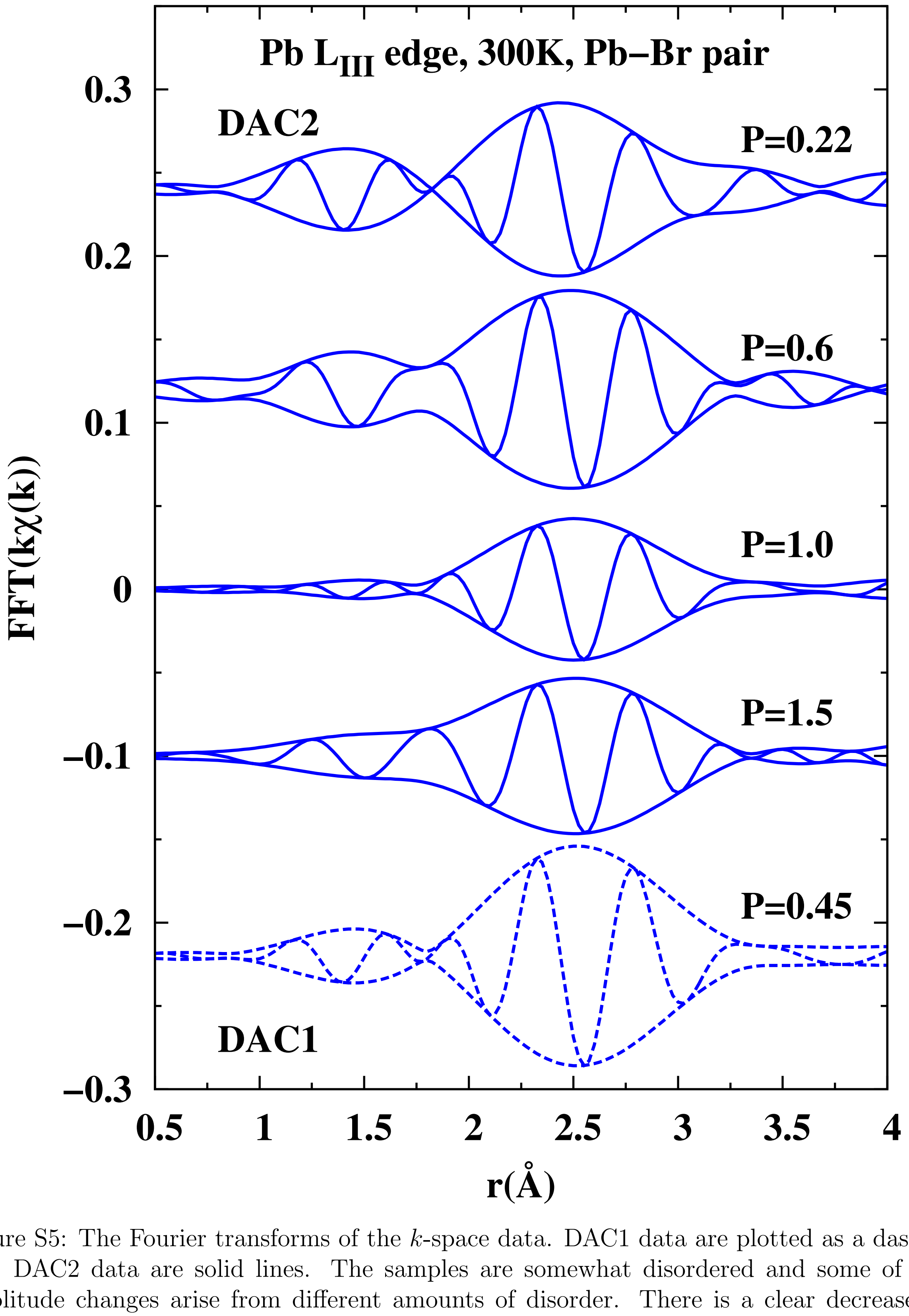

Figure S5: The Fourier transforms of the *k*-space data. DAC1 data are plotted as a dashed line, DAC2 data are solid lines. The samples are somewhat disordered and some of the amplitude changes arise from different amounts of disorder. There is a clear decrease in amplitude at the higher pressures suggesting increased disorder develops with increasing pressure. The Fourier transform range is 4 - 9.7 Å^{-1}.

Bulk modulus calculation

In this section we calculate the bulk modulus of a single $PbBr_6$ octahedron within the corner-sharing inorganic octahedral superstructure of MAPB. Modifying the standard expression for B by considering that $P = -(dU/dV)_T$ we have:

$$B = V \frac{d}{dV} \frac{dU}{dV} \tag{1}$$

The volume of a regular octahedron is given by:

$$V_{oct} = \frac{\sqrt{2}}{3} s^3 \tag{2}$$

where s is the edge length of the octahedron equivalent to $\sqrt{2}r$ with r the Pb-Br bond length.

For a single octahedron we consider the MAPB unit cell shown in Figure S6. Due to the corner-sharing network of octahedra, each unit cell contains one Pb atom and three Br atoms. Therefore, we divide by Z = 4 atoms and get:

$$B = \nu \frac{d}{d\nu} \frac{du}{d\nu} \tag{3}$$

where $\nu = V/4$ is the volume per atom in the $PbBr_3$ octahedra unit cell and u the energy per atom. Then, with $\nu = \frac{1}{3} r^3$ and $\frac{\partial u}{\partial \nu} = \frac{\partial u}{\partial r} \frac{\partial r}{\partial \nu}$,

$$B_{\mathrm{oct}} = \frac{1}{3} r^3 \frac{1}{r^2} \frac{\partial}{\partial r} \frac{1}{r^2} \frac{\partial u}{\partial r} \tag{4}$$

The system is at equilibrium when $r = R$, the Pb-Br distance obtained with EXAFS. Finally, we have:

$$B_{\mathrm{oct}} = \frac{1}{3R} \frac{\partial^2 u}{\partial r^2}\bigg|_{r=R} \tag{5}$$

The spring constant $\frac{\partial^2 u}{\partial r^2}\Big|_{r=R}$ of 1.82 eV/Å^2 is taken from Weadock, et al.,[1] and, with 1.0

eV/Å^2 = 16.02 N/m, B_{oct} = 32.2 GPa.

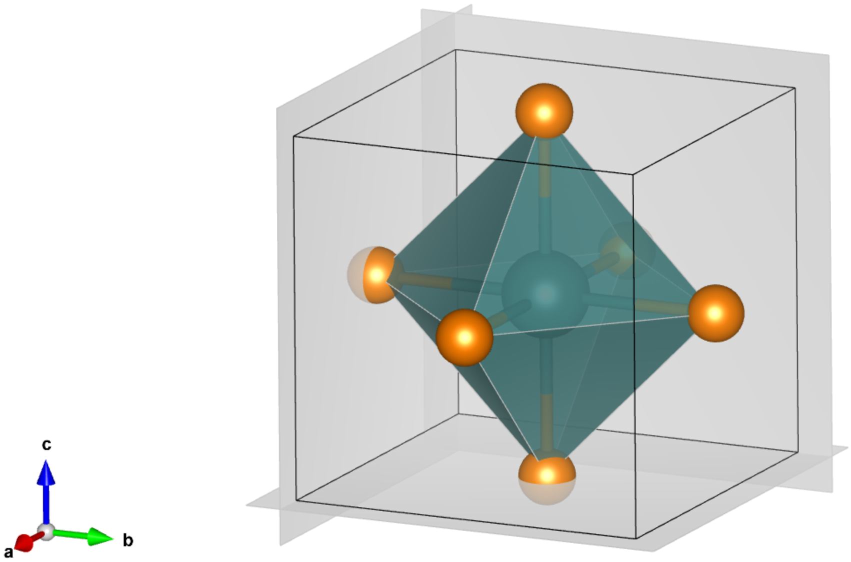

Figure S6: The cubic $Pm\bar{3}$m $MAPbBr_3$ unit cell, centered at Pb (0.5, 0.5, 0.5), consisting of a single $PbBr_6$ octahedron. The shaded planes indicate how each Br atom is split at the cell boundary for a total of Z = 4 atoms per unit cell. The Pb atom is colored blue-green and the Br atoms orange. The C, N, H atoms are omitted for clarity.

References

(1) Weadock, N. J.; MacKeen, C.; Qin, X.; Waquier, L.; Rakita, Y.; Vigil, J. A.; Karunadasa, H. I.; Blum, V.; Toney, M. F.; Bridges, F. Thermal Contributions to the Local and Long-Range Structural Disorder in CH3NH3PbBr3. *PRX Energy* **2023**, *2*, 033004.
